# Advancements in eye movement measurement technologies for assessing neurodegenerative diseases

**DOI:** 10.3389/fdgth.2024.1423790

**Published:** 2024-07-04

**Authors:** Tali G. Band, Rotem Z. Bar-Or, Edmund Ben-Ami

**Affiliations:** Department of Neuroscience, NeuraLight Ltd., Tel Aviv, Israel

**Keywords:** eye tracking, eye movement, neurodegeneration, digital biomarkers, computer vision, machine learning, patient experience

## Abstract

Eye movements have long been recognized as a valuable indicator of neurological conditions, given the intricate involvement of multiple neurological pathways in vision-related processes, including motor and cognitive functions, manifesting in rapid response times. Eye movement abnormalities can indicate neurological condition severity and, in some cases, distinguish between disease phenotypes. With recent strides in imaging sensors and computational power, particularly in machine learning and artificial intelligence, there has been a notable surge in the development of technologies facilitating the extraction and analysis of eye movements to assess neurodegenerative diseases. This mini-review provides an overview of these advancements, emphasizing their potential in offering patient-friendly oculometric measures to aid in assessing patient conditions and progress. By summarizing recent technological innovations and their application in assessing neurodegenerative diseases over the past decades, this review also delves into current trends and future directions in this expanding field.

## 1 Introduction

Neurodegenerative Diseases (NDs) represent a diverse spectrum of conditions characterized by progressive neuronal dysfunction within the Central Nervous System (CNS), potentially culminating in neural cell death. Noteworthy among these disorders are Alzheimer's disease (AD), Parkinson's Disease (PD), and Amyotrophic Lateral Sclerosis (ALS), collectively impacting millions globally each year. For instance, estimates suggest that approximately 4.7 million individuals aged 65 years or older were diagnosed with AD in the United States in 2010 (1), with a global prevalence of 6.1 million for PD in 2016 (2). As the prevalence of NDs correlates with aging demographics (3), projections anticipate a substantial increase in their volume in the coming years.

The clinical presentation of NDs encompasses a wide range of symptoms spanning motor, cognitive, and behavioral domains, exhibiting considerable variability not only between different disorders but also among individuals. This clinical heterogeneity, compounded by the gradual accumulation of symptoms preceding a definitive diagnosis, poses significant challenges to accurate diagnosis. Moreover, many NDs feature a pre-symptomatic phase, which may extend over several years before the onset of apparent clinical symptoms. This pre-symptomatic period holds significant implications for potential preventive interventions and disease-modifying therapies. However, current diagnostic modalities often lack the sensitivity required to detect NDs during this critical phase. Even after diagnosis, uncertainties persist, particularly in the early stages, further complicated by the diverse clinical spectrum encompassed within Parkinson's plus syndromes (4–6). The inadequacy of reliable diagnostic tools, coupled with the inherent subjectivity of clinical evaluation and inter-individual variability, underscores the urgent need for objective biomarkers capable of capturing both motor and cognitive processes (7, 8).

Abnormalities in eye movements are evident in various NDs, including PD (9–12), ALS (13–16), and AD (17–19). Several types of eye movements are affected by neuronal pathology, of which the most prominent are saccades—rapid eye movements that move the line of sight between successive points of fixation (20). Some of these oculomotor abnormalities cannot be observed during a standard clinical examination, and a recording is required to obtain accurate and objective measurements (7, 16, 21). Various oculomotor abnormalities hold promise as potential biomarkers for both diagnosing NDs and monitoring their progression (7, 10, 16, 21). In numerous studies, oculometric measures (OMs) demonstrate temporal reliability and stay consistent over short intervals (22–24).

Some OMs were shown to distinguish between different phenotypes of indications with similar clinical symptoms, providing valuable insights into disease progression and management. For instance, smooth pursuit eye movement features, and specifically gain, were found to be significantly different in the early stages of PD, Progressive Supranuclear Palsy (PSP), Corticobasal syndrome (CBD), and Multiple System Atrophy (MSA), when many clinical symptoms are similar or indistinctive (6, 12, 18).

Although various OMs strongly correlate with several ND acknowledged outcome measures, the optimal OM compositions, and their interpretation remain contingent on the diagnosed disease. For example, in PD patients, correlations have been observed between scores on the MDS-Unified Parkinson's Disease Rating Scale (MDS-UPDRS) and anti-saccade features (25), while ALS patients show similar correlations of anti-saccade latency and error rate with their Revised Amyotrophic Lateral Sclerosis Functional Rating Scale (ALSFRS-R) scores—with additional correlation between smooth pursuit intrusive saccade rate and their ALSFRS-R scores (26, 27). Similarly, patients with AD demonstrate robust correlations between their Mini Mental State Examination (MMSE) scores and specific OMs, namely pro-saccadic latency (28) and micro-saccade lateral bias (19).

In this mini-review, we provide an overview of the current landscape of sensors and techniques for assessing OM abnormalities (Section 2.1) and explore various examination environments and setups facilitating objective oculomotor measurements (Section 2.2). Additionally, we discuss the implications, clinical trends, and anticipated advancements in Section 3.

## 2 Extracting oculometric measures

Accurate gaze estimations in eye-tracking technology are contingent upon several critical parameters to ensure precision while prioritizing patient comfort and ease of use. When considering sample precision, achieving high spatial resolution and addressing system-specific artifacts to ensure measurement accuracy are emphasized. Simplifying setup complexity in terms of device size and complexity, software interfaces, and operator and user interfaces will be considered when accounting for the patient experience. Striving for a non-invasive and user-friendly setup is essential to minimize patient burden, enabling eye movement measurements without necessitating head restraint or other restrictive measures.

### 2.1 Sensors and technology

Theoretically, all OMs may be accurately extracted given a long enough time series of the subject gaze direction with sufficient temporal and spatial resolution. Therefore, the main challenges in the sensing system used for OM extraction are spatial accuracy and sample rate, limiting the OM types that may be extracted using a specific sensor. Pro saccadic latency (the time interval between the stimuli of a saccadic eye movement and the beginning of the actual eyeball movement) values can be as short as 100 ms, and the visual angle amplitude of microsaccades may be as narrow as 0.1 degrees (29). Therefore, OM extraction abilities depend on the sensors’ combination of spatial accuracy and sample rate.

Along with the physical limitations of OM extraction, the advancements in computation capabilities and the reduction in electronic component sizes enabled the development of sensors that are both accurate, affordable, and patient-friendly, led by video imagers accompanied by dedicated computer vision software. Due to these trends of increasing measurement accuracy and smaller equipment size, sensors are now planned to be used more frequently across diverse populations. Therefore, they are required to be as comfortable as possible, with minimal burden on the subjects being examined (30, 31).

#### 2.1.1 Electrooculography

Electrooculography (EOG) entails the strategic placement of electrodes on the periorbital skin to monitor voltage fluctuations corresponding to ocular movements. This technique facilitates the distinct capture of both horizontal and vertical eye movements, even in the absence of any ocular stimulation and when the subjects' eye may be closed (30, 32). While EOG presents distinct advantages in terms of cost-effectiveness and minimal energy consumption compared to alternative eye-tracking modalities, its resolution remains constrained by susceptibility to artifacts, mainly from external sources such as ambient electrical field perturbations. Moreover, physiological artifacts originating from muscular activity, particularly during bodily motions or facial muscle engagement by participants, contribute further to signal distortion (32–34). Nevertheless, with continued research endeavors, substantial potential exists to augment precision and advance the sophistication of EOG technologies.

#### 2.1.2 Scleral coil system

The scleral coil system involves affixing one or two coils onto the ocular globe and quantifying induced voltages resulting from alterations in magnetic fields precipitated by ocular movements. Most scleral coil sensors are shaped as a ring and placed on the sclera surrounding the iris, similar to eye contacts, but often with an additional wire that connects devices external to the eye. Despite the advantageous feature of minimal noise interference, its invasive nature warrants consideration, as its utilization is constrained to a recommended duration of 30 min or less, primarily due to predictable discomforts, including ocular dryness and transient corneal deformities. It is imperative to acknowledge that the presence of the search coil significantly affects select oculomotor parameters, notably saccadic latency and peak velocity (35, 36).

#### 2.1.3 Video oculography

Video Oculography (VOG), a technology grounded in non-invasive video graphics, has garnered increasing attention over the past two decades (37–39). This innovative approach employs one or multiple cameras (monochromatic or multispectral) to carefully examine the gathered data from captured images, seamlessly adaptable through either a head-mounted apparatus or the integration of head-free webcams within computing systems or handheld devices (30).

The fusion of video-based tracking alongside advanced software platforms for the extraction of OMs has recently demonstrated remarkable precision (16, 31, 40, 41). Vigorous endeavors are underway to develop models to augment accuracy and achieve precision levels commensurate with contemporary IR eye trackers, obviating the necessity for supplementary configurations or costly apparatus. Extensive research continues exploring the efficacy of machine learning and neural network architectures in tracking ocular movements (42). Noteworthy among these advancements is the NeuraLight platform, leveraging the video-based NeuraLight Gaze Estimation Model (NLGEM) and the Calibrated Gaze Model (CGM), showcasing equivalence to established references such as the Tobii eye tracker (43, 44). The CGM model capitalizes on visual stimuli for measurements, facilitating ongoing real-time calibration during testing sessions and obviating the requirement for discrete calibration procedures (45).

#### 2.1.4 Infrared eye tracker

A notable advancement in the domain of eye tracking materialized with the advent of infrared (IR) systems. These setups emit infrared light toward the subject's eyes, measuring the reflected light to ascertain the precise location of the pupil's center and to approximate the Point-of-Regard (PoR, the location the subject is looking at) (46, 47). Such systems can be mounted on desktop monitors and laptops, and even integrated into wearable head devices (44, 48). Initially, achieving a stable head orientation was imperative for ensuring measurement accuracy; however, contemporary iterations of eye trackers demonstrate commendable resilience, swiftly recuperating from head movements. Calibration remains a prerequisite for accurate PoR detection within this framework. Despite exhibiting relatively minimal noise levels in comparison to EOG, this methodology still has its susceptibilities, with various artifacts potentially arising from idiosyncratic patient factors, encompassing eyelid morphology, eyelash length, and the utilization of corrective lenses or spectacles (12, 47, 49). Noteworthy is the treatment of IR technology as a distinct entity within the broader purview of VOG video-based gaze systems (31). Within the confines of this mini-review, we opt to delineate IR technology as a discrete entity, underscored by its distinctive attributes. Unlike the conventional camera-based VOG systems, which passively receive data in the visual light spectrum, relying on external light sources, IR technology proffers an active component, thereby imbuing it with an additional layer of functionality. However, similar to VOG methodologies, inherent challenges may emerge when applying IR tracking technique from diverse patient characteristics.

### 2.2 Examination setup

In the past, eye-tracking systems necessitated intrusive measures, demanding physical constraints on the subject's head. Typically, studies relied on visual stimuli presented of a singular monitor, screen, or light array, with participants seated, and their heads immobilized using various means such as chin rests. A visual stimulus would manifest on a screen positioned before them, set at a specific visual height and a viewing distance typically ranging between 60 cm and 70 cm (27, 50). However, strides within the field have introduced non-intrusive configurations, allowing participants unrestricted head movements while upholding precision (51). Ambient factors, including light sources, exert notable influence on the accuracy of gaze detection. Optimal laboratory conditions dictate sound and light isolated rooms to mitigate distractions and keep participants focused on the assigned tasks. Hence, minimizing direct and ambient sunlight, oftentimes achieved by dimming or even extinguishing non-essential lighting sources, proves indispensable. A well-lit environment ensures pupil constriction, consequently enhancing data quality, particularly in scenarios involving variable luminance stimuli (52).

The evolution of physical eye-tracking setups has been remarkable, transitioning from conventional head-mounted apparatuses (48) to the integration of webcams within computing devices or handheld gadgets such as smartphones (53) or tablets (54). This progression facilitates precise measurements without the necessity of head restraining. For instance, in tablet setups, the device screen is oriented vertically, with the camera side facing upward, securely affixed at eye level through the employment of a tablet pole mount. The subject face is positioned at an approximate distance of 45 cm from the screen (55). Ongoing endeavors are directed toward refining head-free tracking capabilities accommodating variations in head positioning, distances, and illumination conditions (56). Challenges specific to this domain, such as “head-gaze correlation overfitting” and “head pose ambiguity,” are diligently addressed in pursuit of better accuracy.

Empirical evidence suggests that the accuracy of measurements obtained solely through smartphone utilization rivals that of dedicated eye-tracking systems. Notably, one study reported a minimal error margin of 0.46 cm on the smartphone screen (equivalent to 0.6–1 degrees of viewing angle), requiring less than 30 s of calibration data per user (42). The potential for enhancing smartphone-based eye-tracking systems through refined calibration methodologies remains palpable (53). Embracing smartphone technology presents a cost-effective alternative to conventional eye-tracking devices, fostering scalability and enabling broader sample sizes in clinical research endeavors (42).

Looking ahead, advancements in virtual reality (VR) technology promise to broaden the scope of eye-tracking applications. Although VR users, particularly those with neurodegenerative diseases, may experience cybersickness (57, 58) or even oculomotor function changes (59), integrating eye-tracking functionalities into VR headsets adds a spatial dimension to stimuli, enriching the interactive experience (5). These technological strides pave the way for more cost-effective and portable equipment, thereby extending the reach of eye tracking beyond traditional laboratory or clinical settings to telemedical homes and external environments. The expanded accessibility enhances the prospect of engaging a more diverse pool of patients and control groups for comparative analyses (31, 49), thereby fostering the accumulation of richer datasets. With increased data availability, the potential for accuracy enhancement and further technological refinement is substantial.

## 3 Discussion

The integration of high-precision gaze-detection systems with accessible setups holds promise for significant scientific advancements in analyzing eye movement and oculometric measurements among patients with NDs (45). These advancements facilitate comfortable examination procedures in clinics as well as remote measurements in patients' homes, assuming access to a computing device equipped with a webcam (e.g., tablet, smartphone, laptop, or desktop computer). Such remote monitoring enables the sampling of a vastly larger number of patients, expanding the training sets of various models and thereby enhancing their accuracy. Higher accuracy and reliability are suggested to expand usage and increase the volumes of the training data, closing a positive feedback loop. Moreover, validated video-based remote OM assessment platforms are expected to reduce costs in pharmaceutical clinical trials and accelerate the usage volume growth, adding diverse demographics and ethnicities and providing positive reinforcement for the projected adaptation of these platforms. Looking ahead, self-operated home-based ND monitoring is a feasible emerging stage in the future ND assessment protocol.

As a field adjusting to recently developed technology, further research is required to study the correlations between ND outcome measures and both traditional and newly developed OMs. However, we surf the front wave of the relevant technologies and develop the ND assessment platforms accordingly. Rapid improvements in computational abilities include complex machine learning and artificial intelligence models, as well as the reduction and acceleration of processing units and data storage devices. In addition, hardware recent developments of imaging sensors in various wavelengths and sensitivities, both eye tracking technologies offer an opportunity to integrate their input with the booming computational power and soon finalize a first validated platform for ND assessment based on eye movements.

Although minimal and preferably seamless, advanced examination setups for eye-movement abnormality measurement present certain challenges. Head-mounted devices are limited in sampling rate, particularly affecting saccade analysis; handheld devices encounter numerous issues, including spatial resolution discrepancies among different cameras, introducing variability in head positions and angles, and variations in distance from the camera (49). These challenges must be addressed to enhance the efficacy of eye-tracking methodologies in ND research.

Future trends in eye-movement assessment for NDs may introduce platforms that continuously measure and extract OMs without pre-defined visual stimuli. Such platforms may be integrated with daily used displays like smartphones or desktop computers or on any future computing device that enables eye tracking, including Virtual Reality (VR) or Augmented Reality (AR) devices. VR/AR devices introduce additional dimension to the apparent visual field (depth), enabling the extraction of unique OMs that are influenced by the depth coordinate of the PoR.

Assessing ND severity and progression using eye-movement abnormality measurements and the development and definition of the OMs that will found these measurements are emerging applications in their booming stage. While initial promising results have already been shown in recent software and hardware studies, recent advancements have introduced a feasible potential for a more affordable and patient-friendly platform for assessing ND condition and progression.
